# An Account of the Practice of One of the Physicians of the Westminster General Dispensary, and of the Western Dispensary, from the 20th of January, to the 20th of February, 1808

**Published:** 1808-03

**Authors:** Samuel Fothergill

**Affiliations:** Southampton-Street, Strand


*An Account of the Practice of one of the Physicians of the Westminster General Dispensary, and of the Western Dispensary, from the 20th of January, to the 20th of February,* 1808.

| | | | |
|---|---|---|---|
| ACUTE DISEASES. | | Acute Diseases of Infants | 9 |
| Catarrh - - | 4 | CHRONIC DISEASES. | |
| Pleurisy - - | 3 | Cough and Dyspnœa | 46 |
| Acute Rheumatism - | 5 | Pulmonary Consumption | 4 |
| Inflammatory sore Throat | 3 | Hæmoptoe - | 3 |
| Measles - - | 12 | Asthma - - - | 2 |
| Fever with Dyspnœa | 4 | Chronic Rheumatism | 14 |

Lumbago

| | | | |
|---|---|---|---|
| Lumbago and Sciatica | 5 | Pyrosis - - | 2 |
| Cephalalgia and Vertigo | 6 | Tumid Abdomen - | 1 |
| Asthenia - - | 12 | Dysure and Gravel - | 3 |
| Dropsy - - - | 5 | Jaundice - - | 2 |
| Constipation - - | 7 | Palpitation - - | 1 |
| Diarrhœa - - | 3 | Cutaneous Eruption - | 4 |
| Gastrodynia - - | 5 | Amenorrhœa - - | 6 |
| Enterodynia - - | 2 | Abortion - - | 2 |
| Dyspepsia - - | 6 | Leucorrhœa - - | 4 |

Much severity and sudden changes of weather have lately been experienced. Illness has consequently been more general than in milder winters, or where the temperature, though colder in degree, is of a more uniform standard. Children and aged people appear to have suffered the most. Many of the former continue to be affected with measles; and some of them have suffered severely with urgent difficulty of breathing, accompanied with considerable frequency of pulse and febrile heat. Several people advanced in years have complained of general debility, chilliness, impaired action of the stomach, and depression of mind. These symptoms are probably occasioned by the depressing power of cold upon an exhausted system, and if neglected, not unfrequently terminate in serous apoplexy, or dropsy; both of which, in many instances, may be prevented by timely and judicious care.

I have recently attended a young artist, who for some time had been affected with occasional and intense pains of the head, preceded by a sense of coldness; vertigo and dimness of sight nearly approaching to blindness, sncceeded with great faintness, which obliged him to desist from his studies, and repose for a considerable space before he was able to proceed in his employment. These symptoms increased to such an alarming degree, that he fully expected each succeeding attack would be fatal, and had resigned himself with much fortitude to his apprehended destiny. I considered the case to be purely nervous; the patient was of a sanguine melancholic temperament, with a fine imagination, ardent in the pursuit of his profession, and his mind agitated by various sources of anxiety. Under this impression, I gave large doses of sal-succini, with asafœtida; urged the necessity of diminishing application to one subject; of varying the mental stimuli, and mingling more in the cheering converse of social social life. This plan has been successful; and the patient is now nearly restored to health and vigour of mind.

In a delicate habit, with refined feelings, and sensibility approaching what has not unaptly been termed morbid, anxiety of mind is very frequent, and not seldom produces serious effects. It interrupts and suspends the repose of sleep, excites febrile action, destroys vital energy, and occasionally induces consumption, and paralysis. These cases are not to be combated by medicine alone; the source of anxiety must be sought for, and the prevailing passion gratified or subdued before we can effect much by the aid of drugs; hence, an acquaintance with the constitution of the mind, and the springs of the human heart, is essential to the philanthropic practitioner, and the avenue to these is seldom opened by the patient till soothed into confidence. From the numerous trifling cases of what are called nervous complaints, it is too common to regard all affections of that nature as trivial; but, now and then, we meet with instances too striking to be mistaken; and sometimes too fatal to be forgotten. Many of these might be saved from destruction: mental derangement is seldom instantaneous; suicide is rarely committed without previous indications of anxiety, or some unusual disturbance of the mental faculties; yet we find people rather employed in removing knives and poison out of the unfortunate sufferer's reach, than solicitous in investigating, and using competent means for eradicating the exciting cause of the malady, which might more frequently be effected; but medical men, in these cases, are generally consulted when the mischief is irremediable.

SAMUEL FOTHERGILL.

*Southampton-Street, Strand.*

